# A Thrombomodulin Mutation that Impairs Active Protein C Generation Is Detrimental in Severe Pneumonia-Derived Gram-Negative Sepsis (Melioidosis)

**DOI:** 10.1371/journal.pntd.0002819

**Published:** 2014-04-24

**Authors:** Liesbeth M. Kager, W. Joost Wiersinga, Joris J. T. H. Roelofs, Onno J. de Boer, Hartmut Weiler, Cornelis van 't Veer, Tom van der Poll

**Affiliations:** 1 Center for Experimental and Molecular Medicine, Academic Medical Center-University of Amsterdam, Amsterdam, The Netherlands; 2 Center for Infection and Immunity Amsterdam (CINIMA), Academic Medical Center-University of Amsterdam, Amsterdam, The Netherlands; 3 Division of Infectious Diseases, Academic Medical Center-University of Amsterdam, Amsterdam, The Netherlands; 4 Department of Pathology, Academic Medical Center-University of Amsterdam, Amsterdam, The Netherlands; 5 Blood Research Institute, Blood Center of Wisconsin, Milwaukee, Wisconsin, United States of America; Mahidol University, Thailand

## Abstract

**Background:**

During severe (pneumo)sepsis inflammatory and coagulation pathways become activated as part of the host immune response. Thrombomodulin (TM) is involved in a range of host defense mechanisms during infection and plays a pivotal role in activation of protein C (PC) into active protein C (APC). APC has both anticoagulant and anti-inflammatory properties. In this study we investigated the effects of impaired TM-mediated APC generation during melioidosis, a common form of community-acquired Gram-negative (pneumo)sepsis in South-East Asia caused by *Burkholderia (B.) pseudomallei*.

**Methodology/Principal Findings:**

(WT) mice and mice with an impaired capacity to activate protein C due to a point mutation in their *Thbd* gene (TM^pro/pro^ mice) were intranasally infected with *B. pseudomallei* and sacrificed after 24, 48 or 72 hours for analyses. Additionally, survival studies were performed. When compared to WT mice, TM^pro/pro^ mice displayed a worse survival upon infection with *B. pseudomallei*, accompanied by increased coagulation activation, enhanced lung neutrophil influx and bronchoalveolar inflammation at late time points, together with increased hepatocellular injury. The TM^pro/pro^ mutation had limited if any impact on bacterial growth and dissemination.

**Conclusion/Significance:**

TM-mediated protein C activation contributes to protective immunity after infection with *B. pseudomallei*. These results add to a better understanding of the regulation of the inflammatory and procoagulant response during severe Gram-negative (pneumo)sepsis.

## Author Summary

Pneumonia and sepsis are conditions in which a procoagulant state is observed, with activation of coagulation and downregulation of anticoagulant pathways, both closely interrelated with inflammation. The protein C (PC) system is an important anticoagulant pathway implicated in the pathogenesis of sepsis. After binding to thrombomodulin (TM), PC is converted into active protein C (APC), mediated via high-affinity binding of thrombin to thrombomodulin (TM) and further augmented via association of the endothelial protein C receptor (EPCR) to the TM-thrombin complex. We studied the role of TM-associated PC-activation during the host response during pneumonia-derived sepsis caused by *Burkholderia (B.) pseudomallei*, the causative agent of melioidosis, a common form of community-acquired Gram-negative (pneumo)sepsis in South-East Asia and a serious potential bioterrorism threat agent. Mice with an impaired capacity to activate protein C displayed a worse survival upon infection with *B. pseudomallei*, accompanied by increased coagulation activation, enhanced lung neutrophil influx and bronchoalveolar inflammation at late time points, together with increased hepatocellular injury. These data further expand the knowledge about the role of the protein C system during melioidosis and may be of value in the development of therapeutic strategies against this dangerous pathogen.

## Introduction

Thrombomodulin (TM, CD141) is a multifunctional transmembrane glycoprotein receptor expressed on the surface of all vascular cells and various hematopoietic cells involved in activation of various parameters of inflammation and coagulation including protein C (PC), thrombin-activatable fibrinolysis inhibitor (TAFI), complement factors and in high mobility group box-1 (HMGB1) [1], [2]. TM plays a pivotal role in the regulation of coagulation via its capacity to activate PC into active protein C (APC), mediated by high-affinity binding of thrombin to TM [3], [4] and further augmented via association of the endothelial protein C receptor (EPCR) to the TM-thrombin complex [3], [4]. Once dissociated from EPCR, APC serves as an anticoagulant by inactivating coagulation factors Va and VIIIa, together with its cofactor protein S [3], [4]. On the other hand, APC has anti-inflammatory, cytoprotective and anti-apoptotic properties through signaling via G-coupled protease activated receptors-1 (PAR-1) [4]. Futhermore, APC may exert anti-inflammatory effects via PAR-3 [5] and involvement of α_3_β_1_, α_5_β_1_, and α_V_β_3_ integrins [6], mechanisms that are in part EPCR-independent.

Ample evidence has shown that severe (pneumo)sepsis is accompanied by both activation of a strong proinflammatory response and increased coagulation activation, inadequate anticoagulation and suppression of fibrinolysis [7], [8]. The interplay between inflammation and blood coagulation is considered to be an essential part of host defense against pathogenic bacteria. Indeed, patients with severe sepsis displayed low levels of PC and APC, which correlated with organ dysfunction and an adverse outcome [9], [10]. Preclinical studies investigated the role of endogenous PC during inflammation and sepsis. Mice with decreased PC levels, due heterozygous deficiency for PC, had more severe disseminated intravascular coagulation, increased fibrin depositions and higher levels of proinflammatory cytokines upon intraperitoneal injection with lipopolysaccharide (LPS) [11], while reduced PC levels in mice with genetically modified (low) PC expression strongly correlated with a survival disadvantage after LPS challenge [12]. Furthermore, inhibition of endogenous PC increased the procoagulant response during *Escherichia coli* peritonitis [13] and H1N1 influenza in mice [14].

Melioidosis is an infectious disease common in Southeast-Asia and Northern-Australia and an important cause of community-acquired pneumonia and sepsis in these areas with mortalities up to 40% despite appropriate antibiotic therapy [15]–[17]. Once a patient is infected by the causative pathogen *Burkholderia (B.) pseudomallei*, this bacterium spreads rapidly throughout the body resulting in many possible disease manifestations, septic shock being the most severe [15], [16]. Additionally, *B. pseudomallei* was recently classified as a ‘Tier 1’ disease agent considered to be an exceptional threat to security [18]. Previous research has demonstrated pronounced coagulation activation in patients with culture-proven septic melioidosis together with downregulation of anticoagulant pathways [10], [19]. In particular, PC levels were markedly decreased in these patients [10], [19], correlating with a worse disease outcome [10]. In the present study, we sought to determine the role of TM and in particular its function in endogenous APC generation, in the host defense during pneumosepsis caused by *B. pseudomallei*.

## Materials and Methods

### Mice

Pathogen-free 10-week old male WT C57BL/6 mice were purchased from Charles River (Maastricht, The Netherlands). TM^pro/pro^ mice were generated as described [20] and backcrossed eight times on a C57BL/6 background. Homozygous mutant TM^pro/pro^ mice, due to a single amino acid substitution (Glu404Pro) in the *Thbd* gene, exhibit a decrease of approximately 1000-fold with respect to PC activation and approximately 100-fold with respect to binding of thrombin at physiologic levels of the enzyme [20]. In addition, TM^pro/pro^ mice produce less than 4% of APC in their alveolar space upon intratracheal administration of PC and thrombin [21]. Mice were maintained at the animal care facility of the Academic Medical Center (University of Amsterdam), according to national guidelines with free access to food and water. The Committee on Use and Care of Animals of the University of Amsterdam approved all experiments.

### Ethics statement

Mice studies were carried out under the guidance of the Animal Research Institute of the Academical Medical Center in Amsterdam (ARIA). All animals were maintained at the animal care facility of the Academic Medical Center (University of Amsterdam), with free access to food and water, according to National Guidelines for the Care and Use of Laboratory Animals, which are based on the National Experiments on Animals Act (Wet op de Dierproeven (WOD)) and the Experiments on Animals Decree (Dierproevenbesluit), under the jurisdiction of the Ministry of Public Health, Welfare and Sports, the Netherlands. The Committee of Animal Care and Use (Dier Experimenten Commissie, DEC) of the University of Amsterdam approved all experiments (Permit number DIX100121-101700)

### Experimental infection and determination of bacterial growth

Experimental melioidosis was induced by intranasal inoculation with *B. pseudomallei* strain 1026b (750 colony forming units (CFU)/50 µL 0.9% NaCl) as previously described [22]–[25]. The number of mice per group used in each experiment is provided in the Figure Legend. For each experiment all mice were infected at the same time point to avoid variance in the bacterial inoculum. For survival experiments mice were checked every 4–6 hours until death occurred for a maximum of 15 days. Sample harvesting and processing and determination of bacterial growth were done as described [22]–[25].

### Cell counts and flow cytometry

Bronchoalveolar lavage fluid (BALF) was obtained as described [24]. Total counts of paraformaldehyde (4%)-fixed BALF cells were measured using a Coulter Counter (Beckman Coulter Inc. Brea, CA). Differential counts were determined by FACS (FACSCalibur, Becton Dickson, San Jose, CA) using directly labeled antibodies against Gr-1 (Gr-1 FITC; BD Pharmingen, San Diego, CA) and F4/80 (F4/80 APC; AbD Serotec, Oxford, UK). Neutrophilic granulocytes were defined according to their scatter pattern and Gr-1 positivity. All antibodies were used in concentrations recommended by the manufacturer.

### Assays

Interleukin (IL)-6, IL-10, IL-12p70, interferon (IFN)-γ, monocyte-chemoattractant protein-1 (MCP-1) and tumor necrosis factor-α (TNF-α) were measured by cytometric bead array (CBA) multiplex assay (BD Biosciences, San Jose, CA) in accordance with the manufacturers' recommendations. Thrombin-antithrombin complexes (TATc; Siemens Healthcare Diagnostics, Marburg, Germany) and D-dimer (Asserachrom D-dimer, Roche Woerden, the Netherlands) were measured with commercially available ELISA kits. Protein levels in BALF were measured using a Bradford-based protein assay (Bio-Rad Laboratories, Hercules, CA). Aspartate aminotranspherase (ASAT) and alanine aminotranspherase (ALAT) were determined with commercial available kits (Sigma-Aldrich, St. Louis, MO), using a Hitachi analyzer (Boehringer Mannheim, Mannheim, Germany) according to the manufacturers' instructions.

### Histology and immunohistochemistry

Paraffin-embedded 4 µm tissue sections were stained with haematoxylin and eosin (H&E) and analyzed for inflammation and tissue damage as described [22]–[25]. Briefly, all slides were coded and scored by a pathologist blinded for the experimental groups. Lung tissues were scored for the following parameters: interstitial inflammation, necrosis, endothelialitis, bronchitis, edema, pleuritis, presence of thrombi and percentage of lung surface with pneumonia. All parameters were rated separately from 0 (condition absent) to 4 (most severe condition). The total histopathological score was expressed as the sum of the scores of the individual parameters, with a maximum of 24. Granulocyte stainings, using fluorescein isothiocyanate-labeled rat-anti-mouse Ly-6G mAb (BD Pharmingen, San Diego, CA) were done as described previously [23]–[25]. Slides were counterstained with methylgreen (Sigma-Aldrich, St. Louis, MO). The total tissue area of the Ly-6G-stained slides was scanned with a slide scanner (Olympus dotSlide, Tokyo, Japan) and the obtained scans were exported in TIFF format for digital image analysis. The digital images were analyzed with ImageJ (version 2006.02.01, National Institutes of Health, Bethesda, MD) and the immunopositive (Ly6G+) area was expressed as the percentage of the total lung surface area.

### Statistical analysis

Data are expressed as box and whisker plots showing the smallest observation, lower quartile, median, upper quartile and largest observation or as medians with interquartile ranges. Comparisons between groups were tested using the Mann-Whitney *U* test. For survival studies Kaplan-Meier analyses followed by Log-rank (Mantel-Cox) test were performed. All analyses were done using GraphPad Prism version 5.01 (GraphPad Software, San Diego, CA). *P*-values<0.05 were considered statistically significant.

## Results

### TM^pro/pro^ mice have a reduced survival during murine melioidosis

**Figure 1:**
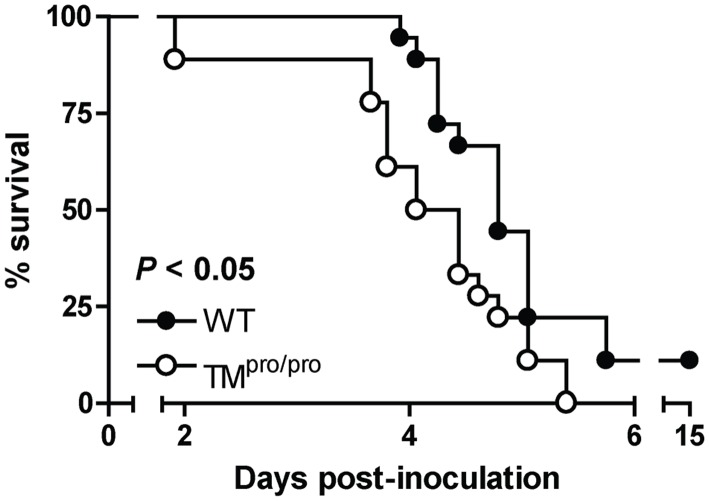
TM^pro/pro^ mice display a reduced survival during murine melioidosis. Mice were inoculated intranasally with 750*B. pseudomallei*. Mortality was assessed every 6 hours, *n* = 16–18 mice per group. Comparison cumulative survival between groups was done by using Kaplan-Meier analysis followed by Log rank (Mantel-Cox) tests.

To explore whether a decreased capacity to generate APC impacts on survival during severe Gram-negative (pneumo)sepsis caused by *B. pseudomallei* we infected TM^pro/pro^ and WT mice with 750 CFU of this bacterium and followed them for 15 days (Figure 1). TM^pro/pro^ mice had an accelerated mortality when compared to WT mice: after 3.8 days already 7 out of 16 TM^pro/pro^ mice (44%) had died, whereas the first WT mice did not die until 3.9 days. After the total observation period, 16 out of 18 WT mice had died (89%), while all TM^pro/pro^ mice had passed away (100%) (*P*<0.05; Figure 1). These results indicate that a reduced capacity to generate APC renders mice more vulnerable for death during Gram-negative (pneumo)sepsis caused by *B. pseudomallei*.

### TM^pro/pro^ mice demonstrate increased coagulation activation after infection with *B. pseudomallei*

**Figure 2:**
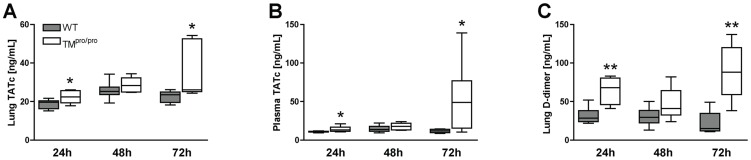
TM^pro/pro^ mice demonstrate increased coagulation activation after infection with *B. pseudomallei*. Mice were inoculated intranasally with 750*B. pseudomallei* and sacrificed after 24, 48 and 72 hours. Coagulation activation in lung homogenates (A) and plasma (B), as reflected by levels of TATc. Lung D-dimer levels in TM^pro/pro^ mice (C). Data are expressed as box and whisker plots showing the smallest observation, lower quartile, median, upper quartile and largest observation. Grey and white boxes represent WT and TM^pro/pro^ mice respectively (*n* = 8 mice/group). *P<0.05 and **P<0.01 for the difference between WT and TM^pro/pro^ mice (Mann-Whitney *U* test).

We have previously shown that in our model of murine melioidosis severe inflammation is associated with marked coagulation activation, which is most prominent at later time points [22]–[25]. To determine whether the increased mortality of TM^pro/pro^ mice was accompanied by alterations in local and systemic coagulation activation of *B. pseudomallei*, we measured levels of TATc, a well-known marker for coagulation activation, in the lungs and systemically in TM^pro/pro^ and WT mice 24, 48 and 72 hours after infection. In accordance with their detrimental phenotype in the survival study, TM^pro/pro^ demonstrated increased coagulation activation, as reflected by elevated pulmonary and plasma levels of TATc at 24 and 72 hours after infection with 750 CFU *B. pseudomallei* intranasally (*P*<0.05 for the differences between WT and TM^pro/pro^ mice, Figure 2A and B). Moreover, when compared to WT mice, TM^pro/pro^ mice had increased lung levels of D-dimer at these time points (*P*<0.01, Figure 2C). These data show that a point mutation in the TM-gene associated with a decreased capacity to generate APC leads to enhanced coagulation activation during Gram-negative (pneumo)sepsis (melioidosis).

### The TM^pro/pro^ mutation has limited impact on bacterial growth and dissemination

**Figure 3:**
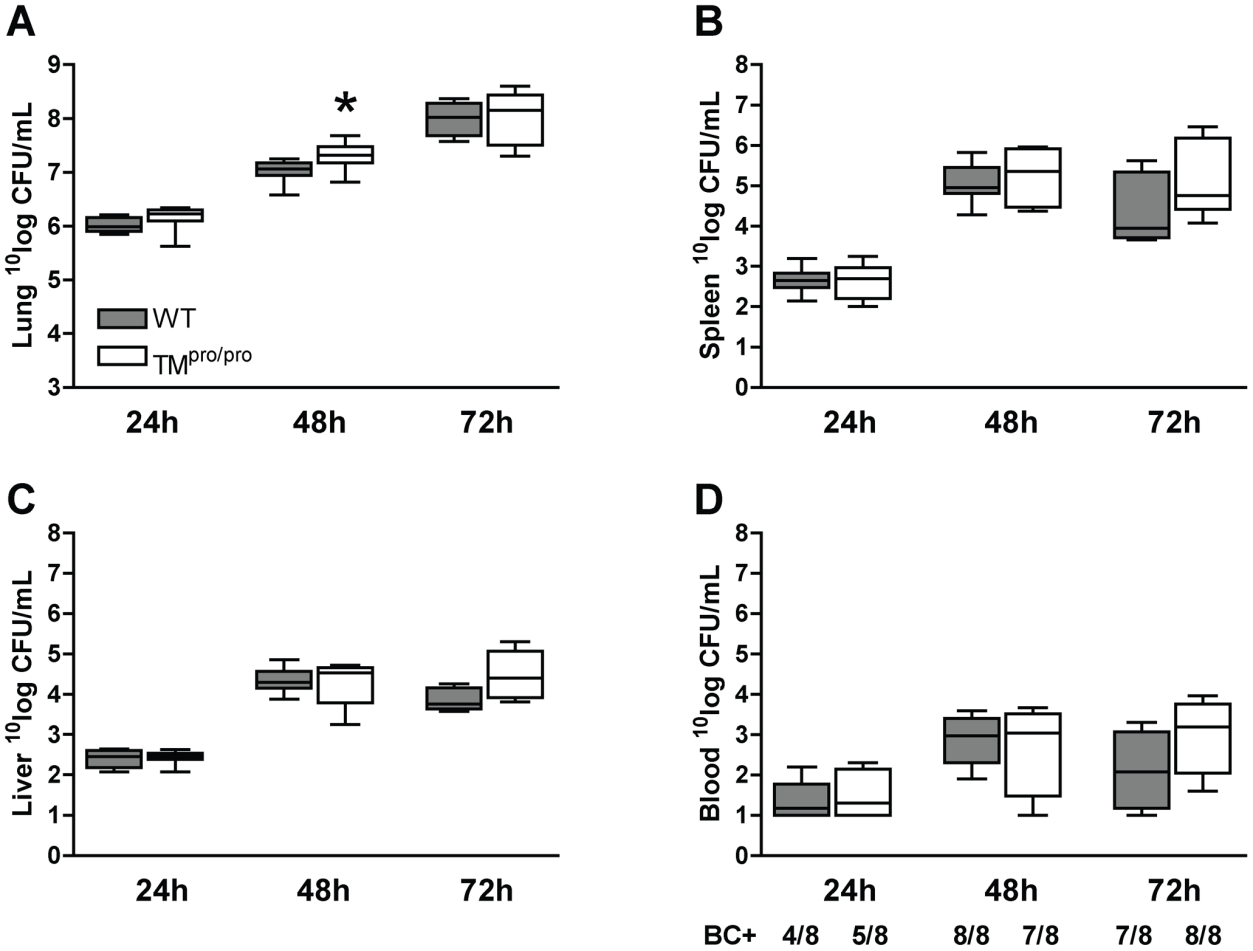
The TM^pro/pro^ mutation has limited impact on bacterial growth and dissemination. Mice were inoculated intranasally with 750*B. pseudomallei* and sacrificed after 24, 48 and 72 hours. Bacterial loads were determined in lung homogenates (A), spleen (B) and liver homogenates (C) and in whole blood (D). Data are expressed as box and whisker plots showing the smallest observation, lower quartile, median, upper quartile and largest observation. Grey boxes represent WT mice, white boxes represent TM^pro/pro^ mice (*n* = 8 mice/group). *P<0.05 for the difference between WT and TM^pro/pro^ mice (Mann-Whitney *U* test). BC+ number of positive blood cultures/total number of mice per group.

Our model of murine melioidosis is associated with marked bacterial growth locally in lungs with subsequent spreading to distant organs [22]–[25]. To determine whether the increased mortality of TM^pro/pro^ mice was accompanied by alterations in the local and systemic growth of *B. pseudomallei*, we examined bacterial loads in the lungs (the primary site of infection), liver, spleen and blood (to evaluate the extent of bacterial dissemination) harvested from TM^pro/pro^ and WT mice 24, 48 and 72 hours after infection with 750 CFU of *B. pseudomallei*. At 48 hours modestly increased bacterial loads were counted in lungs of TM^pro/pro^ mice when compared to WT mice (*P*<0.05, Figure 3A). However, after 72 hours pulmonary bacterial loads of WT and TM^pro/pro^ mice were similar. Furthermore, no differences in bacterial dissemination could be detected: WT and TM^pro/pro^ mice had similar bacterial loads in spleen (Figure 3B), liver (Figure 3C) and blood (Figure 3D) at all time points. These data demonstrate that TM-mediated APC-generation has a modest and temporary effect on local antibacterial defense during severe Gram-negative (pneumo)sepsis.

### TM^pro/pro^ mice exhibit increased lung tissue damage at early time points and increased neutrophil influx in the lungs

**Figure 4:**
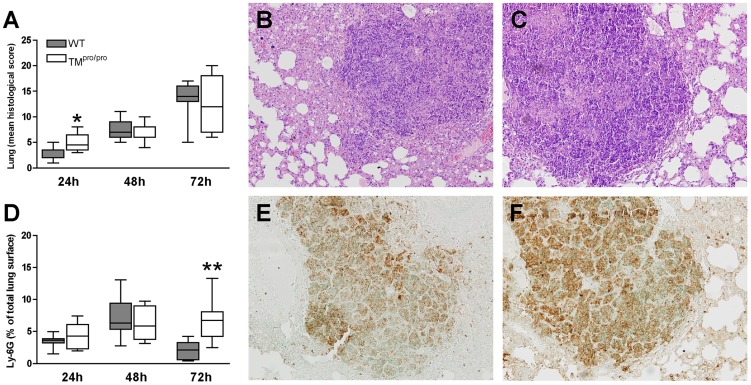
Lung histopathology and neutrophil recruitment. Mice were inoculated intranasally with 750*B. pseudomallei* and sacrificed after 24, 48 and 72 hours. Histopathology scores of WT and TM^pro/pro^ mice infected *B. pseudomallei* (A). Representative photographs of lungs at 72 hours post-inoculation from WT (B) and TM^pro/pro^ mice (C) (H&E staining ×100). Granulocyte influx in the lungs 72 hours after infection, as reflected by the intensity of Ly-6G immunostaining of histopathological slides (D). Representative photographs of Ly-6G immunostaining (original magnification ×100) for granulocytes of WT (E) and TM^pro/pro^ mice (F). Data are expressed as box and whisker plots showing the smallest observation, lower quartile, median, upper quartile and largest observation. Grey boxes represent WT mice, white boxes represent TM^pro/pro^ mice (*n* = 8 mice/group). *P<0.05 and **P<0.01 for the difference between WT and TM^pro/pro^ mice (Mann-Whitney *U* test). CFU colony forming units.

Our murine model of melioidosis is associated with severe lung inflammation and damage [22]–[25]. To analyze whether impaired TM-mediated APC generation would impact hereon, we determined histopathological scores of lungs after infection with *B. pseudomallei*. All mice infected with *B. pseudomallei* had inflammatory lung infiltrates characterized by interstitial inflammation together with necrosis, endothelialitis, bronchitis, edema, thrombi and pleuritis (Figure 4A–C). Twenty-four hours after infection of 750 CFU of *B. pseudomallei* the lung histopathology score (as detailed in the Methods section) was significantly increased in TM^pro/pro^ mice when compared to WT mice (*P*<0.05; Figure 4A–C), while at later time points no differences were seen between both mouse strains. Additionally, we analysed neutrophil recruitment to lung tissue, as it is known that neutrophils play an important role in the host response during melioidosis [16], [17], [26]. For this lung tissues were stained for Ly-6G. Clear neutrophilic infiltrates were seen in both WT and TM^pro/pro^ mice, increasing over time during the course of the experiment. Seventy-two hours after infection, lung tissue of TM^pro/pro^ mice contained significantly more neutrophils than that of WT mice (*P*<0.01, Figure 4D–F). These data suggest that TM-mediated APC generation reduces neutrophil recruitment and lung pathology during severe Gram-negative (pneumo)sepsis.

### Impact of the TM^pro/pro^ mutation on lung and plasma cytokine concentrations after infection with *B. pseudomallei*

Since cytokines and chemokines are important regulators of the inflammatory response to *B. pseudomallei* [16], [17], [27] we measured pulmonary and plasma levels of TNF-α, IL-6, IL-10, IL-12p70, IFN-γ and MCP-1 (Table 1). Interestingly, early (24 hours) after infection of 750 CFU of *B. pseudomallei,* TM^pro/pro^ mice showed reduced IFN-γ levels in both lungs and plasma and decreased IL-12p70 levels in lung homogenates, relative to WT mice. In plasma, these differences remained present at 48 hours after infection. During the late phase of the infection (72 hours) most mediator levels were higher in TM^pro/pro^ mice when compared with WT mice, significantly so for lung IL-12p70 and IL-6 concentrations.

**Table 1:** Cytokine concentrations in lung homogenates and plasma of WT and TM^pro/pro^ mice during murine melioidosis.

	t = 24		t = 48		t = 72	
	WT	TM^pro/pro^	WT	TM^pro/pro^	WT	TM^pro/pro^
**pg/mL**	**Lung homogenates**					
**TNF-α**	1257 (788–1441)	1348 (706–1650)	1875 (1013–2454)	1312 (588–2065)	3593 (2381–4718)	4675 (1030–10000)
**IL-6**	764 (587–990)	935 (395–1022)	1331 (888–1413)	1220 (686–1630)	1802 (802–2447)	3368 (3170–4113)**
**IL-10**	BD	BD	4.0 (3.4–9.1)	3.0 (2.9–7.7)	45 (37–81)	25 (3.2–76)
**IL-12p70**	29 (25–32)	10 (7.9–11)**	24 (21–36)	25 (18–28)	5.6 (4.5–7.5	10 (8.7–17)**
**IFN-γ**	31 (21–35)	18 (15–21)*	21 (15–30)	15 (14–21)	17 (12–24)	20 (16–25)
**MCP-1**	4015 (3469–4371)	3408 (3036–4222)	4053 (3389–5666)	3946 (3334–5307)	9016 (7382–10000)	8402 (5424–10000)
**pg/mL**	**Plasma**					
**TNF-α**	11 (8.6–12)	10 (9.6–12)	29 (16–45)	12 (8.1–19)	47 (34–58)	60 (33–85)
**IL-6**	95 (92–121)	114 (86–131)	776 (206–1598)	251 (166–713)	333 (159–965)	680 (444–1950)
**IL-10**	3.9 (2.8–4.9)	3.4 (3.1–6.3)	2.0 (1.7–2.2)	6.1 (1.5–11)	7.1 (5.6–12)	6.7 (2.7–8.5)
**IL-12p70**	13 (11–14)	10 (7.9–11)	11 (6.3–18)	4.0 (3.4–5.5)**	2.8 (2.0–4.4)	4.0 (3.5–7.1)
**IFN-γ**	27 (24–34)	15 (13–20)**	342 (195–639)	76 (22–139)*	61 (33–106)	142 (86–297)
**MCP-1**	537 (472–659)	395 (376–729)	616 (346–662)	342 (214–562)	479 (155–1014)	687 (503–1696)

Pulmonary and plasma cytokine levels after intranasal infection with 750 CFU of *B. pseudomallei*. Mice were sacrificed 24, 48 or 72 h after infection. Data are expressed as median (interquartile ranges) of *n* = 8 mice per group per time point. BD below detection limits, IFN-γ interferon-γ, IL interleukin, MCP-1 monocyte-chemoattractant protein-1, TNF-α tumor necrosis factor-α.

*P<0.05,

**P<0.01 and

***P<0.001 for WT versus TM^pro/pro^ mice (Mann-Whitney *U* test).

### TM^pro/pro^ mice display increased neutrophil influx and pro-inflammatory cytokine release in the alveolar compartment

**Figure 5:**
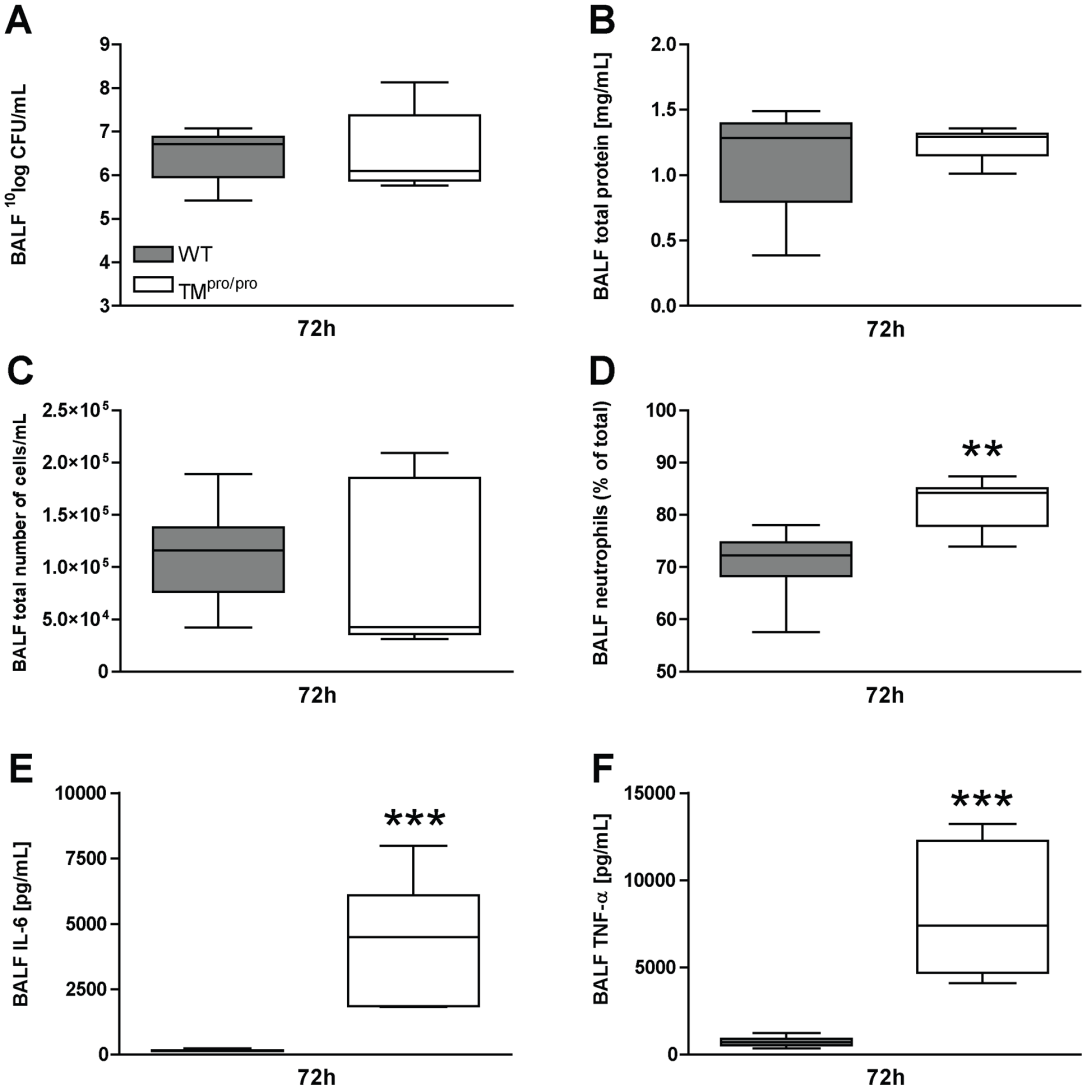
TM^pro/pro^ mice demonstrate an increased inflammatory response in their bronchoalveolar space 72 hours after infection. Mice were inoculated intranasally with 750*B. pseudomallei* and sacrificed after 24, 48 and 72 hours. Bacterial loads (A) in BALF 72 hours after infection with *B. pseudomallei* (A). Total protein content (B), total cell influx (C) and percentages of neutrophils (D) in BALF. Levels of IL-6 (E) and TNF-α (F) in BALF. Data are expressed as box and whisker plots showing the smallest observation, lower quartile, median, upper quartile and largest observation. Grey boxes represent WT mice, white boxes represent TM^pro/pro^ mice (*n* = 8 mice/group). **P<0.01 and ***P<0.001 for the difference between WT and TM^pro/pro^ mice (Mann-Whitney *U* test). BALF bronchoalveolar lavage fluid, CFU colony forming units, IL interleukin, TNF-α tumor necrosis factor-α.

Many studies have demonstrated that severe pneumonia may lead to alveolar damage and subsequent alveolar leakage and release of pro-inflammatory parameters [28], [29]. To determine the impact of impaired APC generation on this extra-vascular, intrabronchial compartment, we determined CFU, protein leakage and parameters of inflammation in BALF 72 hours after inoculation of 750 CFU of *B. pseudomallei*, i.e. shortly before the first deaths occurred and at a time point when lung injury is expected to be at its peak. No differences in bacterial growth (Figure 5A) or total protein content, a marker for alveolar damage (Figure 5B), could be detected in BALF of WT and TM^pro/pro^ mice, nor were there any differences in total cell influx in BALF (Figure 5C). The percentage of neutrophils in BALF of TM^pro/pro^ mice, however, was significantly higher than in WT mice (*P*<0.01; Figure 5D), which is in accordance with the increased neutrophil influx visualized by Ly6-staining of lung tissue. Moreover, BALF levels of the proinflammatory cytokines IL-6 (Figure 5E) and TNF- α (Figure 5F) we significantly increased in TM^pro/pro^ mice when compared to WT mice (*P*<0.001 for both cytokines). These results indicate, that during severe Gram-negative (pneumosepsis) intact TM-mediated APC generation limits the proinflammatory response in the alveolar compartment.

### TM^pro/pro^ mice show enhanced hepatocellular injury

**Figure 6:**
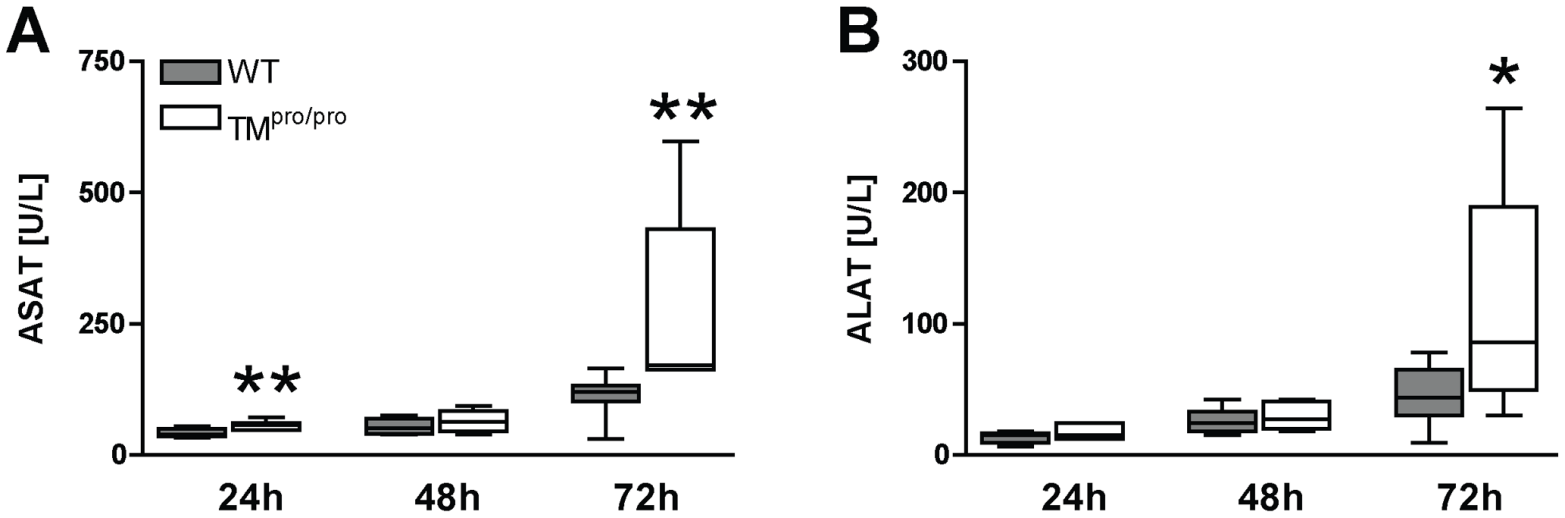
TM^pro/pro^ mice show increased hepatocellular injury during experimental melioidosis. Mice were inoculated intranasally with 750*B. pseudomallei* and sacrificed after 24, 48 and 72 hours. Plasma levels of ASAT (A) and ALAT (B) after infection with *B. pseudomallei*. Data are expressed as box and whisker plots showing the smallest observation, lower quartile, median, upper quartile and largest observation. Grey boxes represent WT mice, white boxes represent TM^pro/pro^ mice (*n* = 8 mice/group). *P<0.05 and **P<0.01 for the difference between WT and TM^pro/pro^ mice (Mann-Whitney *U* test). ASAT aspartate aminotranspherase, ALAT alanine aminotranspherase.

Our model of experimental melioidosis is associated with hepatocellular injury as reflected by elevated plasma levels of transaminases [23], [25]. To obtain insight in the possible role of TM-mediated APC generation herein, we measured ASAT and ALAT in plasma of WT and TM^pro/pro^ mice 24, 48 and 72 hours after infection with 750 CFU of *B. pseudomallei*. Indeed, when compared to WT mice, TM^pro/pro^ mice showed modestly increased levels of plasma ASAT (*P*<0.01 at 24 and 72 hours; Figure 6A) and ALAT (P<0.05 at 72 hours post-infection; Figure 6B). Taken together, intact TM-mediated APC generation seems to protect against hepatocellular injury during experimental melioidosis.

## Discussion

In the present study we sought to investigate the role of TM and in particular its function in endogenous PC activation during melioidosis, a Gram-negative infection often associated with severe pneumonia and sepsis [15], [16]. Melioidosis, as we have demonstrated by our established mouse model, is characterized by gradual growth of bacteria from the lung followed by dissemination to distant body sites, activation of coagulation and inflammation, tissue injury and death, thereby mimicking the clinical scenario of severe (pneumo)sepsis [22]–[25]. Our data show that impaired TM-dependent conversion of PC into APC is associated with enhanced lethality during experimental melioidosis, accompanied by increased coagulation activation, bronchoalveolar inflammation and hepatocellular damage. These data indicate that the capacity to properly activate endogenous PC contributes to protective immunity during experimental melioidosis.

TM is known to play important roles in coagulation and inflammation, that are largely based on its distinct structural domains, including the lectin-like domain, EGF-like repeats, transmembrane domain and short cytoplasmic tail [1], [2]. The EGF-like repeats play a pivotal role in the PC-system via binding of thrombin, thereby increasing the capacity to generate APC a 100-fold [1], [20]. During sepsis, the expression of TM on endothelial cells is downregulated [30], causing impaired APC-generation that may then affect parameters of coagulation and inflammation important for the host response of the infected individual. To answer our research questions, we used genetically modified mice, TM^pro/pro^ mice. In contrast to *Thbd* gene-deficient mice, which die in the embryonic stage [31], TM^pro/pro^ mice develop to term and possess normal reproductive performance [20], but have a decreased endogenous APC synthesis ability when compared to WT mice, as was demonstrated both in the circulation [20] and in the alveolar space [21]. Our data showing increased coagulation activation in TM^pro/pro^ mice, as reflected by increased levels of TATc and D-dimer, are fully in accordance with this. Interestingly, previous studies examining the impact of the TM^pro/pro^ mutation on coagulopathy during experimental (pneumo)sepsis induced by the Gram-positive pathogen *Streptococcus (S.) pneumoniae* or the Gram-negative bacterium *Klebsiella (K.) pneumoniae* or after intranasal administration of *E. coli* LPS failed to show differences in TATc in plasma or BALF between TM^pro/pro^ and WT mice [21]. Similarly, in a model of experimental tuberculosis no differences in lung and plasma TATc were detected between WT and TM^pro/pro^ mice [32]. During systemic endotoxemia TM^pro/pro^ mice were reported to have enhanced fibrin deposition in lungs and kidneys in the presence of unaltered plasma D-dimer concentrations [33]. Clearly, the influence of the TM^pro/pro^ mutation on the procoagulant response depends on the type and extent of the inflammatory stimulus.

Besides its anticoagulant properties TM-activated PC also influences the host immune response during sepsis: APC may exert anti-inflammatory, anti-apoptotic and cell-protective effects by proteolytic cleavage of PAR-1 [3], [4]. Indeed, our data demonstrate that impaired APC generation due to a mutation in the *Thbd* gene resulted in pro-inflammatory effects, as indicated by increased lung pathology at early time points and exaggerated bronchoalveolar inflammation and hepatocellular injury at later time points in TM^pro/pro^ mice. Of interest, the neutrophilic infiltrates measured by Ly-6G staining seem to decrease at 72 hours in WT mice, as opposed to TM^pro/pro^ mice. Remarkably, our results are in contrast with murine models of airway inflammation induced by *S. pneumoniae*, *K. pneumoniae* or LPS, in which no differences in the abovementioned parameters for inflammation were seen between WT and TM^pro/pro^ mice [21]. On the other hand, TM^pro/pro^ mice displayed enhanced diabetic nephropathy, in a model of streptozotocin-induced diabetes mellitus, accompanied by glomerular apoptosis, pointing to a detrimental phenotype when endogenous PC activation is impaired [34], while after induction of lung tuberculosis by *Mycobacterium (M.) tuberculosis* a pro-inflammatory phenotype in TM^pro/pro^ mice was seen [32], comparative to our findings. An obvious explanation for the differences in inflammatory responses observed between these different pathogens and disease conditions is lacking. In contrast to *K. pneumoniae*, both *M. tuberculosis* and *B. pseudomallei* are intracellular organisms, both using the cytosol for survival and escape from anti-bacterial host defense mechanisms. It could be hypothesized that this also influences both generation and inflammatory effects of endogenous APC. Obviously this is area for further research. The coagulation system and APC in particular are of major importance in the host defense against melioidosis, in which both the anti-coagulant and anti-inflammatory function of APC play a major role [24], [35]. It could be hypothesized that during infections with other pathogens such as *K. pneumonia* or *S. pneumonia* the relative contribution of the coagulation system to the host response is of less importance than during hypervirulent *B. pseudomallei* infection in which containment of bacteria at the original site of infection is of utmost importance. In this case it is likely, that deficiency of endogenous APC, as in TM^pro/pro^ mice, has more impact during melioidosis than during infection with other pathogens, which might explain the differences in observed phenotypes between the various pathogens.

In TM^pro/pro^ mice the capacity to generate APC from its precursor protein C is disabled due to a point mutation in the thrombomodulin gene. Therefore, there would be a clear rationale to study exogenously administered APC in our model. However, we have reported very recently that overexpression of APC is detrimental during experimental melioidosis [35]. This is in line with more recent studies in which APC was proven to be ineffective in patients with severe sepsis [36]. Moreover, exogenous recombinant APC has a very short half life, which requires continuous intravenous administration in order to maintain adequate APC levels and to mimic the human situation as much as possible. Obviously, continuous intravenous administration of medication is hard to achieve in freely moving mice.

At late stage infection, shortly before the first deaths occurred, TM^pro/pro^ mice displayed increased local and systemic coagulation activation, increased neutrophil influx and cytokine levels in the lungs, high bacterial loads and -most likely as a consequence thereof- increased end organ damage, as reflected by for example elevated plasma levels of transaminases indicating hepatocellular injury. We therefore hypothesize that the accelerated mortality observed in the TM^pro/pro^ mice is the consequence of a combination of these factors resulting in end-stage multi-organ failure (MOF). In addition, MOF might have induced diffuse intravascular coagulation (DIC) as well, as is often seen in humans with severe sepsis [8]. Indeed, formation of thrombi was observed on histological examination of lung, liver and spleen tissues (data not shown). However, differences between WT and TM^pro/pro^ mice were too small to display any significant differences between groups. Together these data suggest that endogenous APC protects mice against melioidosis induced death by limiting coagulation activation, lung inflammation and MOF.

An important component of the host response to *B. pseudomallei* is the release of proinflammatory cytokines [17], [27], [37]. Clinical studies in melioidosis patients showed elevated serum levels of TNF-α, IL-6 and IFN-γ [27], [37]. The pro-inflammatory cytokine IFN-γ, produced by cytotoxic T-cells and natural killer cells, has an important protective role in early resistance against *B. pseudomallei* infection [38]: administration of a neutralizing monoclonal antibody against IFN-γ was associated with marked increases in bacterial loads in the liver and spleen, together with enhanced lethality [38]. Similarly, inhibition of the production of IL-12, one of the predominant inducers of IFN-γ, resulted in increased mortality in the same model [38]. Interestingly, we found decreased levels of IFN-γ and IL12p70 in TM^pro/pro^ mice early after infection. Although a clear explanation for this observation is lacking, it may in part explain the modestly higher bacterial loads in the lungs of TM^pro/pro^ mice at 48 hours post-infection. While we observed marked differences in pro-inflammatory cytokines between WT mice and TM^pro/pro^ mice, no differences in the anti-inflammatory cytokine IL-10 could be observed. Of note, IL-10 concentrations were very low both in WT mice and TM^pro/pro^ mice during murine septic melioidosis which is in line with earlier reports [39], [40].

Our study also has limitations. It should be noted that there is no consensus in the literature over which mouse strain best models the pathology seen in human melioidosis and both BALB/c and C57BL/6 mice have been used [41]–[43]. BALB/c mice have been thought to be more susceptible for *B. pseudomallei* than C57BL/6 mice, although we and others demonstrated that even after inoculation of a fairly low dose of bacteria (300–750 CFU) C57BL/6 mice develop an acute and severe infection which is lethal in most cases and perfectly mimics acute melioidosis [22], [39], [40], [44]–[46]. The reason for the hypersusceptibility of the BALB/c strain is not known, but Watanabe *et al.* have reported that BALB/c macrophages express lower beta-glucuronidase, in response to levels of the lysosomal enzyme, macrophage-activating lipopeptide-2 (a synthetic TLR2 ligand) and to *E. coli* lipopolysaccharide when compared to C57BL/6 macrophages [47]. In humans, beta-glucuronidase deficiency manifests as ‘Sly syndrome’ or mucopolysaccharidosis type VII. The potential association of the BALB/c mouse with an inherited human disease should prompt caution in the interpretation of experiments conducted using this strain.

The current study identifies TM-mediated APC generation as part of the protective host response during melioidosis and is in accordance with recent evidence from our laboratory showing that inhibition of endogenous PC by specific anti-PC antibodies converts a non-lethal model of experimental melioidosis into a lethal model, associated with increased coagulation activation, severe tissue injury and a strongly increased proinflammatory response [24]. Together these data emphasize the importance of adequate APC levels during melioidosis. As such, administration of recombinant human APC hypothetically could be a promising therapeutic agent in melioidosis. However, in 2012 this drug was withdrawn from the market after negative results from the PROWESS SHOCK trial in sepsis patients [36]. Recombinant soluble TM currently undergoes clinical evaluation as an anticoagulant and anti-inflammatory agent in patients with sepsis [48], [49]. It would be of interest to test the effects of soluble TM in experimental (and clinical) melioidosis.
